# Relationship of cash transfers with risk of overweight and obesity in children and adults: a systematic review

**DOI:** 10.1186/s12889-022-13533-x

**Published:** 2022-06-15

**Authors:** Richard D. Semba, James Manley, Lori Rosman, Nihaal Rahman, Martin W. Bloem

**Affiliations:** 1 Johns Hopkins Center for a Livable Future, Johns Hopkins Bloomberg School of Public Health, Smith Building M015, 400 N. Broadway, Baltimore, MD 21287 USA; 2 Wilmer Eye Institute, Johns Hopkins University School of Medicine, Smith Building M015, 400 N. Broadway, Baltimore, MD 21287 USA; 3 Department of Economics, Towson University, Towson, MD USA; 4 Welch Medical Library, Johns Hopkins University School of Medicine, Baltimore, MD USA; 5 Department of Environmental Health and Engineering, Johns Hopkins Bloomberg School of Public Health, Baltimore, MD USA

**Keywords:** Cash transfer, Obesity, Overweight, Social protection, Systematic review

## Abstract

**Background:**

Cash transfer (CT) programs are an important type of social protection meant to reduce poverty. Whether CT programs increase the risk of overweight and obesity is unclear. The objective was to characterize the relationship between CT programs and the risk of overweight and obesity in children and adults.

**Methods:**

We searched articles in PubMed, Embase, Cochrane, EconLit, Global Health, CINAHL Plus, IBSS, Health & Medical Collection, Scopus, Web of Science, and WHO Global Index Medicus in August 2021. Studies involving CT as the intervention, a control group, body mass index, overweight, or obesity as an outcome, and sample size > 300 were included. The Newcastle–Ottawa Scale was used for quality assessment.

**Results:**

Of 2355 articles identified, 20 met the inclusion criteria. Because of marked heterogeneity in methodology, a narrative synthesis was used to present results. Thirteen of the studies reported that CT programs were associated with a significantly lower risk of overweight and obesity, eight studies showed no significant association, and one study reported a significantly increased risk of obesity in women. Quality assessment showed that most studies lacked sample size and power calculations, validation of exposure, descriptions of non-respondents or those lost to follow-up, and blinded outcome assessment.

**Conclusions:**

Overall, the studies were suggestive that CT programs either have no impact or decrease the risk of overweight and/or obesity in children, adolescents, and adults, but no firm conclusions can be drawn from the available evidence. This review demonstrated limitations in the available studies of CT programs and overweight/obesity.

**Supplementary Information:**

The online version contains supplementary material available at 10.1186/s12889-022-13533-x.

## Introduction

Worldwide, approximately 8.2% of the population, or an estimated 630 million people were living in extreme poverty (< US $1.90/day) in 2019 [1, 2]. An additional ~ 97 million people fell into poverty in 2020 due to the COVID-19 pandemic [3]. Social protection programs, which provide an important safety net for those living in poverty, comprise a wide variety of measures such as cash transfer (CT) programs, school feeding, public works programs, pensions, and unemployment insurance [4]. Social protection is a fundamental part of the United Nations Sustainable Development Goal 1 to end poverty in all its forms everywhere. The goal includes the implementation of nationally appropriate social protection systems and measures for all to achieve substantial coverage of the poor and the vulnerable by 2030 [1].

CT programs are direct, regular and predictable non-contributory payment of money to eligible individuals. CT programs can be either unconditional or conditional. In unconditional CT programs, cash is provided to beneficiaries without any specific obligations to fulfill. In conditional CT programs, cash is provided to beneficiaries under conditions such as health care and education. The World Bank reports that 142 countries have CT programs, of which 70% have unconditional CT programs and 43% have conditional CT programs [5]. The percent of gross national product (GNP) spent on social protection programs in low-, middle-, and high-income countries is 1.5%, 1.6%, and 1.9%, respectively [4]. The number of planned and actual cash CT beneficiaries is 1.8 and 1.5 billion people, respectively [6]. In 2020, over US $1.7 trillion were spent on social protection programs, of which CT programs accounted for 42% of programs, or about US $700 billion [6]. The amount of spending and number of recipients are increasing in the face of the COVID-19 pandemic [6].

CT programs have been shown to reduce the risk of child undernutrition in a recent systematic review and meta-analysis [7]. CT programs targeted to households with young children improved linear growth and reduced stunting in lower- and middle-income countries [7]. The double burden of malnutrition, defined as the simultaneous manifestation of both undernutrition and overweight and obesity, has been increasing worldwide [8]. Whether CT programs increase the risk of the DBM has not been well characterized, mainly because few studies have examined the impact of CT programs on the DBM. However, some studies have described the relationship between CT programs and overweight and/or obesity. Whether CT programs increase the risk of overweight and obesity in children and adults is unclear. Our specific aim was to conduct a systematic review of CT programs and the risk of overweight and obesity in children and adults.

## Materials and methods

For this systematic review, we searched articles in PubMed, Embase, Cochrane, EconLit, Global Health, CINAHL Plus, IBSS, Health & Medical Collection, Scopus, Web of Science, and WHO Global Index Medicus in August 2021 using search terms as shown in Supplementary Table 1. In addition, we hand-searched reference lists of articles identified through the systematic search. CT programs were defined as those programs that provided direct, regular and predictable non-contributory payments of money to eligible individuals. The inclusion criteria for studies were: a control/comparison group was present in the study design, the total sample size was > 300, published after January 1, 1997, written in English, Spanish, or Portuguese, and from the peer-reviewed or gray literature. The exclusion criteria for studies were: pensions, cash-for-work programs, payment-in-kind programs, CT issued in temporary emergency situations or disaster relief, CT programs limited to adults with pre-existing conditions, i.e., human immunodeficiency virus infection, diabetes, etc., or with limited disbursements (< 3).

For children < 5 y, the main outcome measures were overweight (weight-for-height > 2 standard deviation [SD] above the World Health Organization [WHO] growth standards median) and obesity (weight-for-height > 3 SD above the WHO growth standards median) [9]. For children 5–19 y, the main outcome measures were overweight (body mass index [BMI]-for-age > 1 SD above the WHO growth standards median) and overweight (BMI-for-age > 2 SD above the WHO growth standards median) [9]. For adults, the main outcome measures were body weight as a continuous variable, BMI as a continuous variable, overweight (BMI ≥ 25 kg/m^2^) and obese (BMI ≥ 30 kg/m^2^) [10].

Articles from each database search were transferred into EndNote (EndNote 20, Clarivate, Philadelphia, PA, USA), and duplicates were eliminated. Unique references were uploaded into Covidence systemic review software (Veritas Health Innovation, Melbourne, Australia) for title/abstract screening, full-text screening, and finally data extraction of the included studies. Screening and data extraction were conducted by two independent reviewers (RDS, NR). The PRISMA (Preferred Reporting Items for Systematic Reviews and Meta-Analyses) flow diagram was used to summarize the methods [11].

Quality assessment was conducted using the Newcastle–Ottawa scale (NOS) for cohort studies and a modified NOS for cross-sectional studies [12]. The NOS for cohort studies involves evaluation of three factors by giving stars or no stars for: (1) selection (maximum 4 stars), based upon the representativeness of the exposed cohort, the selection of the non-exposed cohort, the ascertainment of exposure, and a demonstration that the outcome of interest was not present at the beginning of the study; (2) comparability of the exposed and nonexposed cohorts, based upon the study design or analysis controlling for confounders (maximum 2 stars); (3) outcome (maximum 3 stars), based upon the method of assessment, whether the follow-up was long enough for the outcome to occur, and the adequacy of follow-up of the cohorts. The modified NOS for cross-sectional studies involves the evaluation of three factors: (1) selection (maximum 4 stars), based upon the representativeness of the sample, the sample size, characterization of non-respondents, and ascertainment of exposure; (2) comparability (maximum 2 stars) based upon comparability of the outcome groups with controlling of confounders; (3) outcome (maximum 3 stars), based upon outcome assessment and statistical testing. The NOS gives a maximum total of 9 stars for each study. NOS assessment of each study was conducted by two independent reviewers (RDS, NR). A consensus discussion was used to reach a final agreed-upon rating for each study.

## Results

**Fig. 1:**
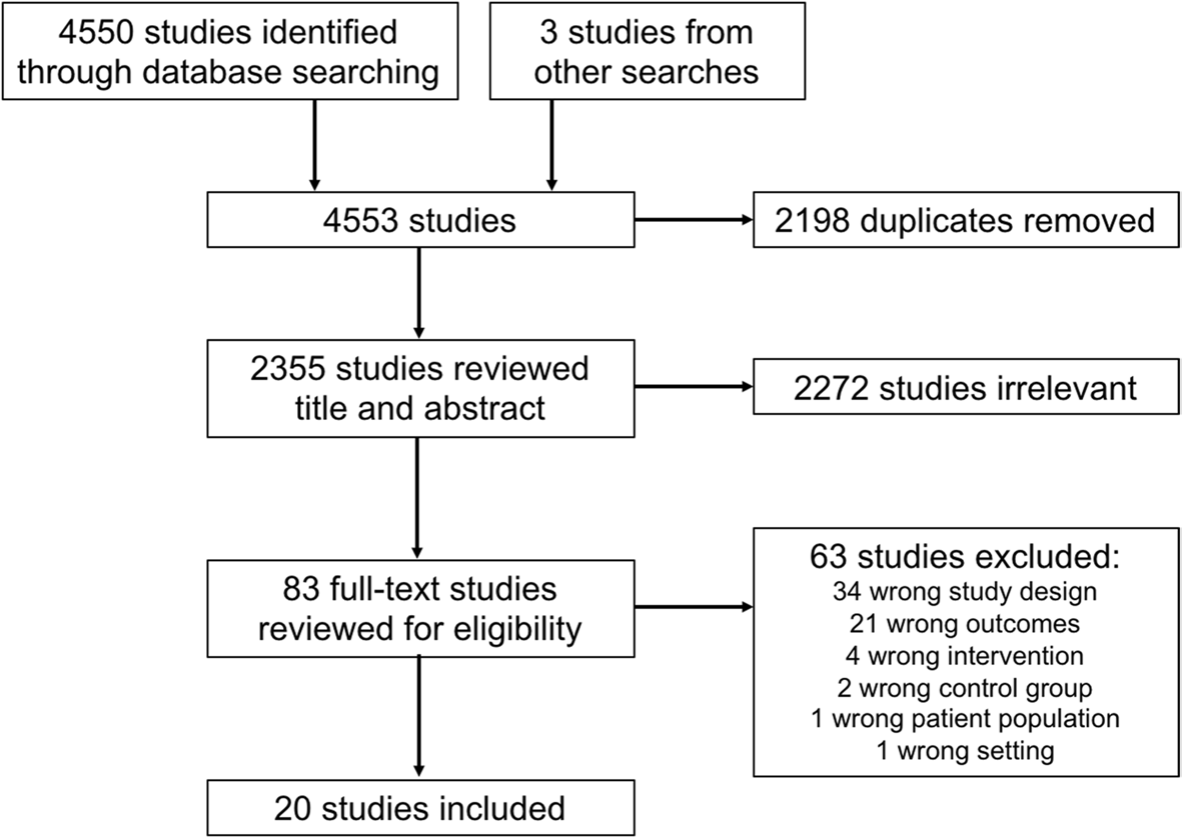
PRISMA flow diagram of studies evaluating the association between CT programs and overweight and/or obesity. Abbreviation: PRISMA, Preferred Reporting Items for Systematic Reviews and Meta-Analysis

The initial search of 11 databases yielded 4550 references. There were 2355 unique references after removing duplicates. Seventeen references fit the inclusion/exclusion criteria, and three additional references that fit the inclusion/exclusion were identified outside of the systematic search. A summary of the review and reasons for excluding studies are shown in the PRISMA flow chart in Fig. 1. The location, study design, methods, outcomes, and results of the twenty eligible studies are summarized in Table 1, grouped by children or adults as the target population. There were eleven studies that examined the relationship between CT programs and child overweight and/or obesity [13–23], eight studies that examined the relationship between CT programs and adult overweight and/or obesity [24–31], and one study that characterized the relationship between CT programs and overweight and/or obesity in both children and adults [32]. The location of most of the studies was in Latin America and the Caribbean [13–19, 22, 24–30, 32]. Of the different major CT programs, there were four studies of *Bolsa Família* in Brazil [13–16], four studies of *Oportunidades *in Mexico [22, 27, 29, 30], two studies of *Familias en Acción* in Colombia [17, 32], and two studies of *Juntos *in Peru [18, 24]. The relationship of CT programs with BMI, overweight and/or obesity, was described in studies from South Africa [21], Japan [20], Canada [31], and the United States (Alaska) [23]. Fourteen of the studies focused on conditional CT programs [13–19, 22, 24, 27–30, 32], and six studies described unconditional CT programs [20, 21, 23, 25, 26, 31].

**Table 1:** Summary of twenty studies reporting the relationship of CT programs with body weight, body mass index, overweight, and obesity

Country	Program	Type	Year (s)	Study population	Study design	Methods	Total (n)	Program (n)	Control (n)	Duration of exposure	Findings	Reference
**Children and adolescents**												
Brazil	*Bolsa Família*	CCT	2006–2007	Children < 5 y; 6 largest municipalities in Maranhāo state; overall prevalence overweight 6.7%	Cross-sectional	State of Maranhāo Health Care Study 2006–2007 data; multivariable logistic regression	1214	not stated	not stated	not stated	Overweight Treatment prevalence ratio (95% CI) 1.0 (0.5, 1.7) (*P* = 0.91)	13
Brazil	*Bolsa Família*	CCT	2013	Children 7–16 y; beneficiaries of National School Meals Program in Guariba, Sāo Paulo state	Cross-sectional	Crude chi-square analyses	409	160	249	not stated	Overweight Treatment: 21.9% Control: 23.3% Obese Treatment: 16.9% Control: 28.9% (*P* < 0.05)	14
Brazil	*Bolsa Família*	CCT	not stated	Children 9 y; 4^th^ graders in Belo Horizonte, Minas Gerais state	Cross-sectional	Data from larger project on food and nutrition education; two-stage cluster sampling; crude chi-square analyses	319	118	201	not stated	Overweight Treatment: 36.3% Control: 30.7% (*P* = 0.59)	15
Brazil	*Bolsa Família*	CCT	2008–2009	Adolescents 10–19 y in Northeast region and Southeast region	Cross-sectional	Family Budget Survey 2008–2009 data; propensity score matching used to identify controls; average treatment effect on the treated (ATT)	4408	1858 (Northeast) 346 (Southeast)	1858 (Northeast) 346 (Southeast)	not stated	Overweight Northeast region: ATT 0.015 (SE 0.015) (n.s.) Southeast region: ATT -0.042 (SE 0.036) (*P* < 0.05)	16
Colombia	*Familias en Acción*	CCT	2002–2006	Children 2–6 y	Longitudinal	Program evaluation data from 2002–2003 and 2005–2006; difference-in-difference (DD) impact estimates calculated	2874	1290	1584	3–4 years	Overweight Treatment OR 1.30 (95% CI 0.83, 2.03) (n.s.) Obese Treatment OR 0.56 (95% CI 0.20, 1.53) (n.s.)	17
Peru	*Juntos*	CCT	2002–2006	Children 7–8 y	Longitudinal; non-participants vs participants < 2 y duration vs participants ≥ 2 y duration	Young Lives Study data; propensity score matching used to identify controls; average treatment effect on the treated (ATT); results described for treatment ≥ 2 y	338	169	169	> 2 years	Overweight baseline Treatment: 37.5% Control: 37.9% (*P* = 0.91) follow-up Treatment: 16.6% Control: 24.9% (*P* = 0.06) Stratified by sex, overweight in girls: ATT -0.22 pp, 95% CI -42.5, -2.7 pp (*P* = 0.03) overweight in boys: n.s., ATT not stated	18
Dominican Republic	*Solidaridad*	CCT	2010	Children < 5 y	Cross-sectional	Social Protection Survey 2010 data; propensity score matching used to identify controls; average treatment effect on the treated (ATT)	2358	1179	1179	not stated	Overweight and obesity ATT -7.0 to -8.7 pp (*P* < 0.05)	19
Japan	*Jido teate*	UCT	2016	Children, grades 1, 5, 8	Cross-sectional	Kochi Child Health Impact of Living Difficulty Study 2016 data; propensity score matching used to identify controls; multivariable logistic regression	434	217	217	not stated	Overweight Treatment: OR 0.51 (95% CI 0.29, 0.91) (*P* = 0.024)	20
South Africa	Child Support Grant	UCT	2012	Children, 5–14 y	Cross-sectional	National Income Dynamics Study, wave 3 data; crude chi-square	6951	5227	1724	not stated	Overweight Treatment: 14.8% Control: 18.0% Obese Treatment: 8.6% Control: 11.8% (*P* < 0.001)	21
Mexico	*Oportunidades*	CCT	1998–2003	Adolescents, 15–21 y, rural areas	Longitudinal	Analysis took advantage of random phase-in of CCT; fuzzy regression discontinuity design; effect of CCT on outcomes calculated as local average treatment effect (LATE); program duration averaged 4 y	2036	not stated	not stated	not stated	Overweight LATE Women -0.137 (0.302) Men 0.069 (0.055) Obesity LATE Women -0.322 (0.157)* Men 0.132 (0.163) (*P < 0.01)	22
USA	Alaska Permanent Fund Dividend (PFD)	UCT	2009–2011	Children age 3 y	Longitudinal	Alaska Longitudinal Child Abuse and Neglect Linkage Project data; obesity age 3 y main outcome;	885	not stated	not stated	3 years	For each $1000, OR 0.69 (*P* < 0.01) for obesity; equivalent to reducing the average probability by 5.2%	23
**Country**	**Program**	**Type**	**Year (s)**	**Study population**	**Study design**	**Methods**	**Total (n)**	**Program (n)**	**Control (n)**		**Findings**	**Reference**
**Adults**												
Peru	*Juntos*	CCT	2007–2013	Mothers	Cross-sectional	Demographic and Health Survey data collected annually 2007–2013; individual and district level analyses; propensity score matching used to identify controls; generalized linear models	5143 individual 24,242 district	not stated	not stated	not stated	Overweight Individual level analysis Prevalence ratio 1.06 (95% CI 0.98, 1.15), (*P* = 0.17) District level analysis Prevalence ratio 0.94 (95% CI 0.90, 0.98), (*P* < 0.001)	24
Mexico	Non-contributory pension	UCT	2007–2008	Adults > 70 y, rural areas, 7 states	Longitudinal	Adults with 11 months exposure to treatment; discontinuity regression approach; 4023 adults	4023	not stated	not stated	11 months	BMI Treatment -0.059 kg/m^2^ (*P* = 0.48)	25
Mexico	Non-contributory pension	UCT	2008–2009	Adults > 70 y in Yucatan state	Longitudinal	Valladolid city, treatment group; Motul city, control group; difference-in-difference (DD) impact estimates calculated	1650	1146	504	6 months	DD of means (SEM) BMI 0.111 (0.120) (n.s.) Overweight -0.037 (0.025) (n.s.) Obese 0.020 (0.018) (n.s.)	26
Mexico	*Oportunidades*	CCT	2002, 2005–2006, 2009–2012	Adults, representative sample of Mexican population at national, rural–urban, and regional level	Longitudinal	Mexican Family Life Survey data; CCT participants (235 stayed in program, 192 left program) and non-participants; propensity score matching used to identify controls; triple difference-in-difference (DDD) impact estimates calculated; average treatment effect on the treated (ATT)	7131	427	6704	variable, up to 10 years	BMI DDD estimate ATT -1.43 kg/m^2^ (*P* < 0.05)	27
Colombia	*Familias en Acción*	CCT	2002, 2006	Women ≥ 18 y	Longitudinal	Surveys conducted in 2002 and 2006; difference-in-difference (DD) impact estimates calculated	2073	1238	835	4 years	BMI Treatment β = 0.25 (95% CI 0.03, 0.47 (*P* = 0.03) Overweight Treatment OR 1.06 (95% CI 0.90, 1.26) (*P* = 0.46) Obesity Treatment OR 1.27 (95% CI 1.03, 1.57) (*P* = 0.03)	28
Mexico	*Oportunidades*	CCT	2003	Adults from rural areas of 7 states	Cross-sectional	Adults receiving CCT for 3.5–5 y compared with newly recruited control group; propensity score matching used to identify controls; ordinary least squares regression	6343	5280	1063	not stated	Overweight Treatment: 59.24% Control: 63.04% (*P* = 0.03) Obesity Treatment: 20.28% Control: 25.31% (*P* < 0.001)	29
Mexico	*Oportunidades*	CCT	2003–2005	Women, 18–49 y, from rural communities in southern and eastern Mexico	Longitudinal	Community randomized controlled intervention trial in 235 communities; CT vs food basket vs control; 23 month duration; difference-in-difference (DD) impact estimates calculated	1507	786	721	mean 14 months	Body weight DD estimate (SEM) 0.4 (0.2) (*P* < 0.05) CT vs control Stratified by normal, overweight, and obese categories at baseline, treatment significantly increased body weight only in women already obese at baseline	30
Canada	Universal Child Care Benefit	UCT	2001–2014	Adults, aged 25–49	Cross-sectional	Canadian Community Health Survey data; treatment group with youngest child 1–5 y, control group with youngest child 6–11 y; difference-in-difference (DD) impact estimates calculated	217,002	107,108	109,984	not stated	BMI Mothers Treatment -0.467 kg/m^2^ (*P* < 0.01) Fathers Treatment -0.075 kg/m^2^ (n.s.) Overweight Mothers Treatment -0.054 (*P* < 0.01) Fathers Treatment 0.007 (n.s.) Obese Mothers Treatment -0.019 (*P* < 0.05) Fathers Treatment -0.009 (n.s.)	31
**Country**	**Program**	**Type**	**Year (s)**	**Study population**	**Study design**	**Methods**	**Total (n)**	**Program (n)**	**Control (n)**		**Findings**	**Reference**
**Both children and adults**												
Colombia	*Familias en Acción*	CCT	2010	Children < 5 y; mothers 18–49 y	Cross-sectional	Demographic and Health Survey data; analysis based upon four household (HH) typologies: normal HH, *n* = 4200 (no stunting or obesity in children, mother normal BMI); underweight HH, *n* = 1250 (at least 1 child stunted, mother underweight); overweight HH, *n* = 5085 (at least 1 child obese, mother overweight/obese or normal); dual-burden HH, *n* = 713 (at least 1 child stunted, mother overweight/obese); 11,248 households in analysis	11,248 households	not stated	not stated	not stated	Treatment Underweight typology OR 0.8 (95% CI 0.7, 1.0) (*P* < 0.10) Overweight typology OR 0.9 (95% CI 0.8, 1.0) (*P* < 0.10) Dual-burden typology OR 0.9 95% CI 0.7, 1.1) (n.s.)	32

The NOS assessment of the studies are shown in Table 2. Eleven studies used a cross-sectional design [13–16, 19–21, 24, 29, 31, 32]. Nine studies used a longitudinal cohort design [17, 18, 22, 23, 25–28, 30].

**Table 2:** Newcastle–Ottawa Scale assessment of studies

**a. Cross-sectional studies**										
**REFERENCE**	**COUNTRY**	**REPRESENTATIVENESS**				**COMPARABILITY**	**OUTCOME**			**TOTAL STARS**
		**representativeness of the sample**	**sample size**	**non-respondents**	**ascertainment of exposure**	**comparability of outcome groups based upon design or analysis**	**assessment of outcome**		**statistical test**	
13	Brazil	⋆	0	0	⋆	⋆	0		0	3
14	Brazil	⋆	0	0	⋆	0	0		⋆	3
15	Brazil	⋆	0	0	⋆	0	0		⋆	3
16	Brazil	⋆	0	0	⋆	⋆⋆	0		⋆	4
24	Peru	⋆	0	0	⋆	⋆⋆	0		⋆	5
32	Colombia	⋆	0	⋆	⋆	⋆⋆	0		⋆	6
29	Mexico	⋆	0	0	0	⋆⋆	0		⋆	4
19	Dominican Republic	0	0	0	⋆	⋆	0		0	2
21	South Africa	⋆	0	0	⋆	0	0		0	2
31	Canada	⋆	0	0	0	⋆	⋆		⋆	4
20	Japan	⋆	0	⋆	⋆	⋆⋆	⋆		⋆	7
**b. Cohort studies**										
**REFERENCE**	**COUNTRY**	**SELECTION**				**COMPARABILITY**	**OUTCOME**			**TOTAL** **STARS**
		**representativeness of exposed cohort**	**selection of non-exposed cohort**	**ascertainment of exposure**	**demonstration that outcome of interest not present at start of sutdy**	**comparability of cohorts based upon design or analysis**	**assessment of outcome**	**follow-up long enough for outcome to occur**	**adequacy of follow-up of cohorts**	
22	Mexico	⋆	⋆	⋆	0	0	0	⋆	0	4
30	Mexico	⋆	⋆	⋆	⋆	⋆⋆	0	⋆	⋆	8
27	Mexico	⋆	⋆	⋆	⋆	0	0	⋆	⋆	6
25	Mexico	0	⋆	0	0	0	0	⋆	0	2
26	Mexico	⋆	⋆	⋆	⋆	⋆⋆	0	⋆	⋆	8
28	Colombia	⋆	⋆	⋆	⋆	⋆⋆	0	⋆	0	7
17	Colombia	⋆	0	⋆	⋆	⋆⋆	0	⋆	0	6
18	Peru	⋆	⋆	⋆	⋆	⋆	0	⋆	0	6
23	USA	⋆	⋆	0	⋆	⋆	0	⋆	0	5

Of the cross-sectional studies, five studies received 2–3 stars, four studies received 4–5 stars, and two studies received 6–7 stars. Of the cohort studies, one study received 2 stars, two studies received 4–5 stars, four studies received 6–7 stars, and two studies received 8 stars. The mean score of the twenty studies was 4.75 stars. Nearly all of the cross-sectional studies were representative of the specific population that was the focus of the study. Ascertainment of exposure was mainly self-report through interviews with participants. Few of the cross-sectional studies provided sample size and power calculations. Most of the studies did not describe the characteristics of non-respondents. Nearly all the cross-sectional studies used anthropometry to assess outcomes, but no studies reported that the study team members who conducted anthropometry were blinded to the exposure assessment. Most of the cross-sectional studies had comparable control groups and adjusted for covariates in the outcomes analyses. Most of the cohort studies were representative of the specific population that was the focus of the study and selected non-exposed controls from the same population. Ascertainment of exposure was by self-report through interviews in most studies.

**Fig. 2:**
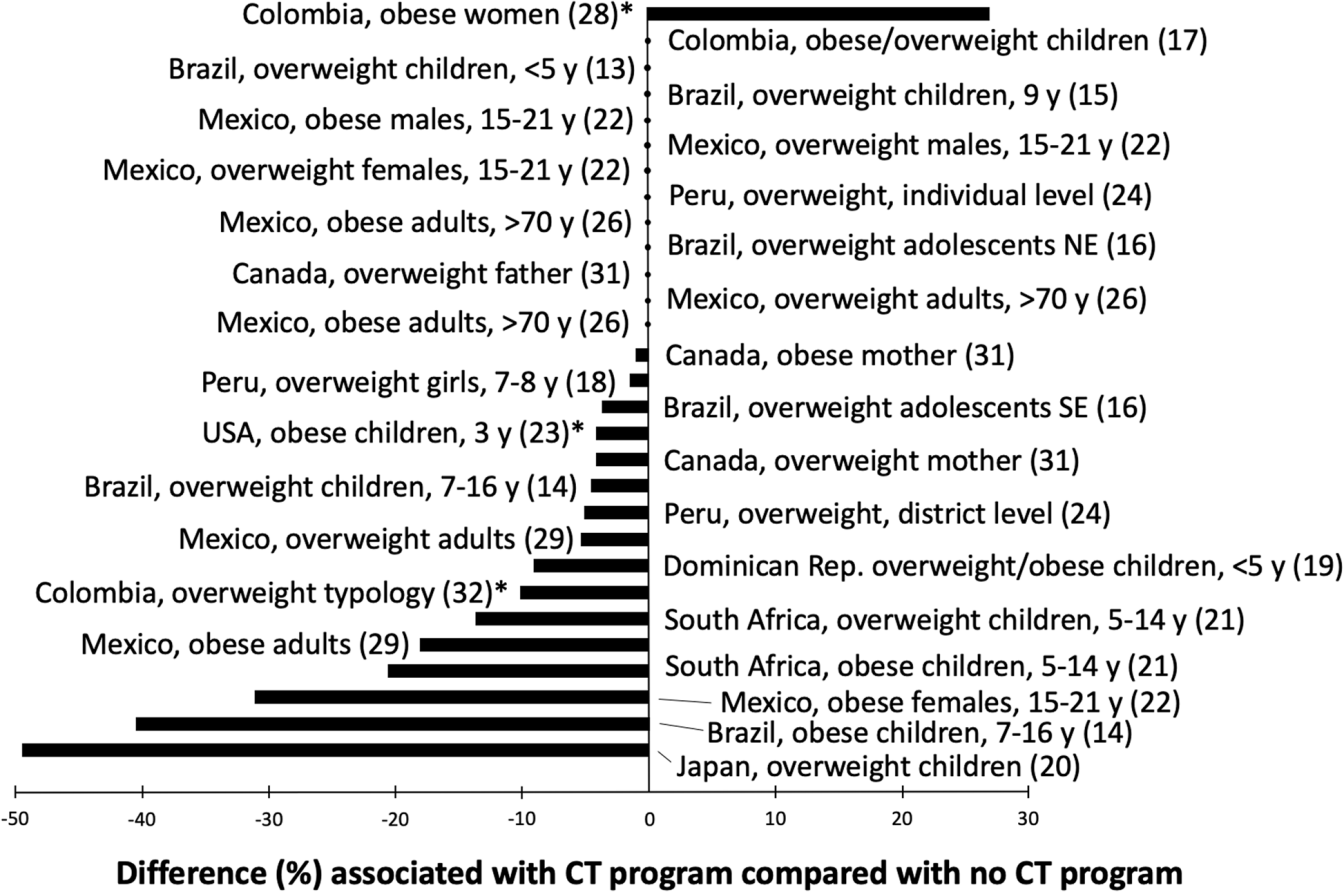
The association between CT programs and overweight and/or obesity as categorical outcomes in fourteen studies. Results that were statistically significant are shown as horizontal bars. Results showing no significant difference between CT programs and overweight or obesity are shown as a dot on the vertical line. Abbreviations: *SE* southeast, *NE* northeast. *Studies that report OR

Given the great deal of heterogeneity in target study populations, study design, statistical methods, CT programs, and outcomes (Table 1), a meta-analysis was not conducted. The relationship of CT programs with overweight and/or obesity as categorical outcomes in seventeen studies is summarized in Fig. 2. One study showed a significantly higher OR for obesity in women > 18 y whose household participated in the CT program in Colombia [28]. Thirteen studies showed that CT programs were significantly associated with lower overweight and/or obesity in children, adolescents, or adults compared with controls [14, 16, 18–24, 26, 29, 31, 32]. Eight studies showed no significant association between CT programs and overweight and/or obesity in the specific target groups that were studied [13, 15–17, 22, 24, 26, 31]. There were three studies that reported the outcome as a continuous variable. In a study from Mexico, the CT program was not associated with a significant change in BMI in adults > 70 y [25]. Another study reported that the CT program was associated with a significant decrease in BMI in a nationally representative sample of adults in Mexico [27]. Among women, aged 18–49 y participating in a community-randomized, controlled intervention trial in rural Mexico, those receiving CT had a significant increase in body weight, especially women who were already obese at baseline [30].

## Discussion

The present review shows that the impact of CT programs on overweight and obesity in both children and adults is not conclusive due to the limited number of studies and mixed results regarding the direction of the association. Thirteen of the twenty studies showed that CT programs were significantly associated with a lower risk of overweight and obesity, while eight studies reported no significant associations. Only one study showed that a CT program was significantly associated with an increased risk of obesity, and the risk was found in women. Overall, the results are suggestive that CT programs either have no impact or decrease the risk of overweight and/or obesity in children, adolescents, and adults, but due to the small number and heterogeneity of studies, no firm conclusions can be drawn from the available evidence.

The CT programs included in this review varied considerably by location, recipients, cash amount, and other characteristics as shown in Supporting Table 2 [33–37]. Most of the conditional CT programs included the present review are located in Latin America and the Caribbean. There was only one study of the association of CT programs with overweight and obesity from Africa [21], although there are currently over forty CT programs in Africa [6]. No CT programs from Asia were identified through the systematic search in the present study.

Overall, the quality of the studies as assessed by the NOS not high, with an average score of 4.75 out of a 9 star rating. The quality evaluation of the studies using the NOS assessment revealed several weaknesses in study design that could be addressed in future research conducted with CT programs, including provision of sample size and power calculations, using measures to ensure blinding of study team members who are measuring the outcomes using anthropometry, describing the characteristics of subjects who are lost to follow-up or refuse participation, and validating the measure of exposure.

A recent meta-analysis showed that CT programs have a small but significant impact on reducing stunting by 2.1% [7]. Another recent review of experimental or quasi-experimental studies showed the CT programs increased birthweight, although the number of studies was small [38]. The double burden of malnutrition, which is the simultaneous manifestation of both undernutrition and overweight and obesity, can occur on the individual, household, and population level [8]. CT programs appear to reduce undernutrition [7, 38], and overall, the twenty studies in the present analysis do not provide strong support for the idea that CT programs increase the risk of overweight and obesity.

The present study was limited to CT programs because these programs represent a major proportion of social protection expenditures worldwide [6]. Other types of social protection, such as school feeding programs, food supplements, and cash-for-work were not included, as the programmatic implications are quite different from CT programs. The present study did not examine the pathway between the increase in household income through CT programs and body mass index or body weight. Factors in the pathway include nutrition education, growth monitoring, household spending, and food expenditures, such as spending on sugar-sweetened beverages [39].

CT programs continue to evolve worldwide, with some accelerated changes due to the COVID-19 pandemic [6]. The CT program in Mexico, most recently known as *Prospera* and formerly *Oportunidades*, was considered a model for CT programs worldwide [40]. *Prospera *was abruptly terminated by the government in 2019 and replaced with an unconditional CT program [41]. The removal of health and education conditions for the CT program in Mexico resulted in a large drop in attendance at health centers and layoffs of frontline healthcare workers [41]. Concerns have been raised that removal of conditions from the CT program in Mexico will adversely affect civic participation among the poor [42]. The role of health conditions of conditional CT programs and the risk of overweight and obesity is not clear and could be addressed in future studies.

Worldwide, CT programs are becoming a preferred form of food assistance, as many countries are shifting from food vouchers and food transfers to CT [43]. CT have a favorable impact upon food consumption and dietary diversity and are more cost-efficient than food-based interventions [44]. Digital payments have improved speed and transparency of CT programs and have achieved deeper financial inclusion [6]. In the age of COVID-19, digital payments have also reduced person-to-person exposure and minimized health risks [6].

Obesity and overweight are important risk factors for chronic diseases such as hypertension [45], diabetes [46], and cardiovascular disease [47]. Thus, CT programs, through their potential impact on overweight and obesity, have the potential to affect long-term health of millions of participants worldwide. CT programs are considered the most important social safety net for social protection programs [4]. Despite the large expenditure on CT programs worldwide (~ US $700 billion in 2020) [6], there are only a limited number of studies that used a randomized controlled design to examine the impact of CT on nutritional outcomes [48].

In conclusion, there were a limited number of studies that described the relationship between CT programs and the risk of overweight and obesity. Overall, CT programs appear to have either no impact or a decreased risk of overweight and/or obesity in children, adolescents, and adults. Due to the small number of studies, heterogeneity of studies, and limited quality of studies, no firm conclusions can be drawn from the available evidence. Further work is needed to characterize the relationship of CT programs with overweight and obesity using more rigorous methodology and inclusion of distal outcomes such as hypertension, diabetes mellitus, and cardiovascular disease.

## Supplementary Information

**Additional file 1: Supplementary Table 1. **Databases and Search Terms used for the Systematic Review.

**Additional file 2: Supporting Table 2. **Characteristics of specific cash transfer programs.

## Data Availability

All data generated during this study are included in the published article.
