# Supplementary material for: Relationship of cash transfers with risk of overweight and obesity in children and adults: a systematic review
Source: BMC Public Health. 2022 Jun 15;22:1190. doi: 10.1186/s12889-022-13533-x (PMC9198205; doi:10.1186/s12889-022-13533-x)
Supplement: Supplementary file 1 — Additional file 1: Supplementary Table 1. Databases and Search Terms used for the Systematic Review. [file 12889_2022_13533_MOESM1_ESM.docx]

**Supplementary Table 1. Databases and Search Terms used for the Systematic Review**

**PubMed**

**Date run: 8Aug2021: 489 results**

| #1 | **"Reimbursement, Incentive"[Mesh]** **OR "Financial Support"[Mesh:NoExp]** **OR "Token Economy"[Mesh]** OR “cash allowance*” [tw] OR “cash assistance” [tw] OR “cash benefit*” [tw] OR “cash measure*” [tw] OR “cash payment*” [tw] OR “cash transfer*” [tw] OR “cash-based approach*” [tw] OR “cash-based assistance” [tw] OR “cash-based programming” [tw] OR “cash-based response*” [tw] OR “financial assistance” [tw] OR “financial benefit*” [tw] OR “financial measure*” [tw] OR “financial allowance*” [tw] OR “financial payment*” [tw] OR “monetary benefit*” [tw] OR “monetary transfer*” [tw] OR “monetary allowance*” [tw] OR “monetary payment*” [tw] OR “money allowance*” [tw] OR “money transfer*” [tw] OR “money voucher*” [tw] OR “multi-purpose cash” [tw] OR “payment measure*” [tw] OR "pay for performance"[tw] OR "financial incentive*"[tw] OR "economic incentive*"[tw] OR "monetary incentive*"[tw] OR "monetary reimbursement*"[tw] OR "Reimbursement Incentive*"[tw] OR "voucher program*"[tw] OR "voucher incentive*"[tw] |
| --- | --- |
| #2 | **“overweight” [Mesh] OR “obesity” [Mesh] OR “body mass index” [Mesh] OR “body weight” [Mesh] OR “waist circumference” [Mesh] OR “adiposity” [Mesh] OR “waist-to-hip ratio” [Mesh] OR “skinfold thickness” [Mesh]** **OR “weight gain” [Mesh]** OR “overweight” [tw] OR “overnutrition” [tw] OR "over nutrition"[tw] OR “obesity” [tw] OR “obese” [tw] OR “body mass index” [tw] OR “BMI” [tw] OR “body weight*”[tw] OR “waist circumference*” [tw] OR “waist-to-hip ratio*” [tw] OR “waist-to-height ratio*” [tw] OR “skinfold thickness” [tw] OR "skin fold thickness"[tw] OR "skin fold measurement*"[tw] OR "skinfold measurement*"[tw] OR “weight gain*” [tw] OR “adiposity” [tw] |
| #3 | #1 AND #2 |

**Embase .com**

**Date run: 8Aug2021: 1,553 results**

| #1 | **'cash transfer'/exp OR 'cash transfer program'/exp OR 'economic incentive'/exp** OR (“cash allowance*” OR “cash assistance” OR “cash benefit*” OR “cash measure*” OR “cash payment*” OR “cash transfer*” OR “cash-based approach*” OR “cash-based assistance” OR “cash-based programming” OR “cash-based response*” OR “financial assistance” OR “financial benefit*” OR “financial measure*” OR “financial allowance*” OR “financial payment*” OR “monetary benefit*” OR “monetary transfer*” OR “monetary allowance*” OR “monetary payment*” OR “money allowance*” OR “money transfer*” OR “money voucher*” OR “multi-purpose cash” OR “payment measure*” OR "pay for performance" OR "financial incentive*" OR "economic incentive*" OR "monetary incentive*" OR "monetary reimbursement*" OR "reimbursement Incentive*" OR "voucher program*" OR "voucher incentive*"):ab,ti,kw |
| --- | --- |
| #2 | **'obesity'/exp OR 'body mass'/exp OR 'body weight'/exp OR 'waist circumference'/exp OR 'waist hip ratio'/exp OR 'skinfold thickness'/exp OR 'body weight gain'/exp** OR (“overweight” OR “overnutrition” OR "over nutrition" OR “obesity” OR “obese” OR “body mass index” OR “BMI” OR “body weight*” OR “waist circumference*” OR “waist-to-hip ratio*” OR “waist-to-height ratio*” OR “skinfold thickness” OR "skin fold thickness" OR "skin fold measurement*" OR "skinfold measurement*" OR “weight gain*” OR “adiposity”):ab,ti,kw |
| #3 | #1 AND #2 |

**Cochrane**

**Date run: 8Aug2021: (~Cochrane Reviews: 80, Cochrane Protocols: 6, Trials: 254)**

| #1 MeSH descriptor: [Reimbursement, Incentive] explode all trees  #2 MeSH descriptor: [Financial Support] explode all trees  #3 MeSH descriptor: [Token Economy] explode all trees  #4 ("cash allowance" OR "cash allowances" OR "cash assistance" OR "cash benefit" OR "cash benefits" OR "cash measure*" OR "cash measures" OR "cash payment*" OR "cash payments" OR "cash transfer*" OR "cash transfers" OR "cash-based approach*" OR "cash-based approaches" OR "cash-based assistance" OR "cash-based programming" OR "cash-based response*" OR "cash-based responses" OR "financial assistance" OR "financial benefit*" OR "financial benefits" OR "financial measure*" OR "financial measures" OR "financial allowance" OR "financial allowances" OR "financial payment" OR "financial payments" OR "monetary benefit" OR "monetary benefits" OR "monetary transfer" OR "monetary transfers" OR "monetary allowance" OR "monetary allowances" OR "monetary payment" OR "monetary payments" OR "money allowance" OR "money allowances" OR "money transfer" OR "money transfers" OR "money voucher" OR "money vouchers" OR "multi-purpose cash" OR "payment measure" OR "payment measures" OR "pay for performance" OR "financial incentive" OR "financial incentives" OR "economic incentive" OR "economic incentives" OR "monetary incentive" OR "monetary incentives" OR "monetary reimbursement" OR "monetary reimbursements" OR "reimbursement Incentive" OR "reimbursement incentives" OR "voucher program" OR "voucher programs" OR "voucher incentive" OR "voucher incentives")  #5 {OR #1-#4}  #6 MeSH descriptor: [Overweight] explode all trees  #7 MeSH descriptor: [Obesity] explode all trees  #8 MeSH descriptor: [Body Mass Index] explode all trees  #9 MeSH descriptor: [Body Weight] explode all trees  #10 MeSH descriptor: [Waist Circumference] explode all trees  #11 MeSH descriptor: [Adiposity] explode all trees  #12 MeSH descriptor: [Waist-Hip Ratio] explode all trees  #13 MeSH descriptor: [Skinfold Thickness] explode all trees  #14 MeSH descriptor: [Weight Gain] explode all trees  #15 ("overweight" OR "overnutrition" OR "over nutrition" OR "obesity" OR "obese" OR "body mass index" OR "BMI" OR "body weight" OR "body weights" OR "waist circumference" OR "waist circumferences" OR "waist-to-hip ratio" OR "waist-to-hip ratios" OR "waist-to-height ratio" OR "waist-to-height ratios" OR "skinfold thickness" OR "skin fold thickness" OR "skin fold measurement" OR "skin fold measurements" OR "skinfold measurement" OR "skinfold measurements" OR "weight gain" OR "weight gains" OR "adiposity")  #16 {OR #6-#15}  #17 #5 AND #16 |
| --- |

**EconLit (Ebsco)**

**Date run: 8Aug2021: 45 results**

| S1 | TX ( ( ("cash allowance*" OR "cash assistance" OR "cash benefit*" OR "cash measure*" OR "cash payment*" OR "cash transfer*" OR "cash based approach*" OR "cash based assistance" OR "cash based programming" OR "cash based response*" OR "financial assistance" OR "financial benefit*" OR "financial measure*" OR "financial allowance*" OR "financial payment*" OR "monetary benefit*" OR "monetary transfer*" OR "monetary allowance*" OR "monetary payment*" OR "money allowance*" OR "money transfer*" OR "money voucher*" OR "multi purpose cash" OR "payment measure*" OR "pay for performance" OR "financial incentive*" OR "economic incentive*" OR "monetary incentive*" OR "monetary reimbursement*" OR "reimbursement Incentive*" OR "voucher program*" OR "voucher incentive*") ) ) |
| --- | --- |
| S2 | TX ( ( ("overweight" OR "overnutrition" OR "over nutrition" OR "obesity" OR "obese" OR "body mass index" OR "BMI" OR "body weight*" OR "waist circumference*" OR "waist-to-hip ratio*" OR "waist-to-height ratio*" OR "skinfold thickness" OR "skin fold thickness" OR "skin fold measurement*" OR "skinfold measurement*" OR "weight gain*" OR "adiposity") ) ) |
| S3 | S1 OR S2 |

**Global Health (OVID)**

**Date run: 8Aug2021: 205 results**

| #1 | ("cash allowance*" OR "cash assistance" OR "cash benefit*" OR "cash measure*" OR "cash payment*" OR "cash transfer*" OR "cash based approach*" OR "cash based assistance" OR "cash based programming" OR "cash based response*" OR "financial assistance" OR "financial benefit*" OR "financial measure*" OR "financial allowance*" OR "financial payment*" OR "monetary benefit*" OR "monetary transfer*" OR "monetary allowance*" OR "monetary payment*" OR "money allowance*" OR "money transfer*" OR "money voucher*" OR "multi purpose cash" OR "payment measure*" OR "pay for performance" OR "financial incentive*" OR "economic incentive*" OR "monetary incentive*" OR "monetary reimbursement*" OR "reimbursement Incentive*" OR "voucher program*" OR "voucher incentive*").mp. |
| --- | --- |
| #2 | **exp overweight/ or exp obesity/ or exp body mass index/ or exp body weight/** OR ("overweight" OR "overnutrition" OR "over nutrition" OR "obesity" OR "obese" OR "body mass index" OR "BMI" OR "body weight*" OR "waist circumference*" OR "waist-to-hip ratio*" OR "waist-to-height ratio*" OR "skinfold thickness" OR "skin fold thickness" OR "skin fold measurement*" OR "skinfold measurement*" OR "weight gain*" OR "adiposity").mp. |
| #3 | 1 AND 2 |

**CINAHL Plus (Ebsco)**

**Date run: 8Aug2021: 333 results**

| S1 | (MH "Reimbursement, Incentive") OR (MH "Financial Support") |
| --- | --- |
| S2 | TI ( ("cash allowance*" OR "cash assistance" OR "cash benefit*" OR "cash measure*" OR "cash payment*" OR "cash transfer*" OR "cash based approach*" OR "cash based assistance" OR "cash based programming" OR "cash based response*" OR "financial assistance" OR "financial benefit*" OR "financial measure*" OR "financial allowance*" OR "financial payment*" OR "monetary benefit*" OR "monetary transfer*" OR "monetary allowance*" OR "monetary payment*" OR "money allowance*" OR "money transfer*" OR "money voucher*" OR "multi purpose cash" OR "payment measure*" OR "pay for performance" OR "financial incentive*" OR "economic incentive*" OR "monetary incentive*" OR "monetary reimbursement*" OR "reimbursement Incentive*" OR "voucher program*" OR "voucher incentive*") ) OR AB ( ("cash allowance*" OR "cash assistance" OR "cash benefit*" OR "cash measure*" OR "cash payment*" OR "cash transfer*" OR "cash based approach*" OR "cash based assistance" OR "cash based programming" OR "cash based response*" OR "financial assistance" OR "financial benefit*" OR "financial measure*" OR "financial allowance*" OR "financial payment*" OR "monetary benefit*" OR "monetary transfer*" OR "monetary allowance*" OR "monetary payment*" OR "money allowance*" OR "money transfer*" OR "money voucher*" OR "multi purpose cash" OR "payment measure*" OR "pay for performance" OR "financial incentive*" OR "economic incentive*" OR "monetary incentive*" OR "monetary reimbursement*" OR "reimbursement Incentive*" OR "voucher program*" OR "voucher incentive*") ) OR SU ( ("cash allowance*" OR "cash assistance" OR "cash benefit*" OR "cash measure*" OR "cash payment*" OR "cash transfer*" OR "cash based approach*" OR "cash based assistance" OR "cash based programming" OR "cash based response*" OR "financial assistance" OR "financial benefit*" OR "financial measure*" OR "financial allowance*" OR "financial payment*" OR "monetary benefit*" OR "monetary transfer*" OR "monetary allowance*" OR "monetary payment*" OR "money allowance*" OR "money transfer*" OR "money voucher*" OR "multi purpose cash" OR "payment measure*" OR "pay for performance" OR "financial incentive*" OR "economic incentive*" OR "monetary incentive*" OR "monetary reimbursement*" OR "reimbursement Incentive*" OR "voucher program*" OR "voucher incentive*") ) |
| S3 | S1 OR S2 |
| S4 | (MH "Obesity+") OR (MH "Body Mass Index") OR (MH "Body Weight+") OR (MH "Waist Circumference") OR (MH "Waist-Hip Ratio") OR (MH "Skinfold Thickness") OR (MH "Weight Gain+") OR (MH "Overnutrition") |
| S5 | TI ( ("overweight" OR "overnutrition" OR "over nutrition" OR "obesity" OR "obese" OR "body mass index" OR "BMI" OR "body weight*" OR "waist circumference*" OR "waist-to-hip ratio*" OR "waist-to-height ratio*" OR "skinfold thickness" OR "skin fold thickness" OR "skin fold measurement*" OR "skinfold measurement*" OR "weight gain*" OR "adiposity") ) OR AB ( ("overweight" OR "overnutrition" OR "over nutrition" OR "obesity" OR "obese" OR "body mass index" OR "BMI" OR "body weight*" OR "waist circumference*" OR "waist-to-hip ratio*" OR "waist-to-height ratio*" OR "skinfold thickness" OR "skin fold thickness" OR "skin fold measurement*" OR "skinfold measurement*" OR "weight gain*" OR "adiposity") ) OR SU ( ("overweight" OR "overnutrition" OR "over nutrition" OR "obesity" OR "obese" OR "body mass index" OR "BMI" OR "body weight*" OR "waist circumference*" OR "waist-to-hip ratio*" OR "waist-to-height ratio*" OR "skinfold thickness" OR "skin fold thickness" OR "skin fold measurement*" OR "skinfold measurement*" OR "weight gain*" OR "adiposity") ) |
| S6 | S4 OR S5 |
| S7 | S3 AND S6 |

**International Bibliography of the Social Sciences (IBSS) (Proquest)**

**Date run: 8Aug2021: 47 results**

| #1 | MAINSUBJECT.EXACT("Cash payments") OR MAINSUBJECT.EXACT("Payments") OR MAINSUBJECT.EXACT("Monetary incentives") OR noft(("cash allowance*" OR "cash assistance" OR "cash benefit*" OR "cash measure*" OR "cash payment*" OR "cash transfer*" OR "cash based approach*" OR "cash based assistance" OR "cash based programming" OR "cash based response*" OR "financial assistance" OR "financial benefit*" OR "financial measure*" OR "financial allowance*" OR "financial payment*" OR "monetary benefit*" OR "monetary transfer*" OR "monetary allowance*" OR "monetary payment*" OR "money allowance*" OR "money transfer*" OR "money voucher*" OR "multi purpose cash" OR "payment measure*" OR "pay for performance" OR "financial incentive*" OR "economic incentive*" OR "monetary incentive*" OR "monetary reimbursement*" OR "reimbursement Incentive*" OR "voucher program*" OR "voucher incentive*")) |
| --- | --- |
| #2 | MAINSUBJECT.EXACT("Overweight") OR MAINSUBJECT.EXACT("Obesity") OR MAINSUBJECT.EXACT("Body mass index") OR noft(("overweight" OR "overnutrition" OR "over nutrition" OR "obesity" OR "obese" OR "body mass index" OR "BMI" OR "body weight*" OR "waist circumference*" OR "waist-to-hip ratio*" OR "waist-to-height ratio*" OR "skinfold thickness" OR "skin fold thickness" OR "skin fold measurement*" OR "skinfold measurement*" OR "weight gain*" OR "adiposity")) |
| #3 | #1 AND #2 |

**Health & Medical Collection (Proquest)**

**Date run: 8Aug2021: 353 results**

| #1 | MAINSUBJECT.EXACT("Cash payments") OR MAINSUBJECT.EXACT("Payments") OR MAINSUBJECT.EXACT("Monetary incentives") OR MESH.EXACT.EXPLODE("Financial Support") OR MESH.EXACT.EXPLODE("Token Economy") OR MESH.EXACT.EXPLODE("Reimbursement, Incentive") OR noft(("cash allowance*" OR "cash assistance" OR "cash benefit*" OR "cash measure*" OR "cash payment*" OR "cash transfer*" OR "cash based approach*" OR "cash based assistance" OR "cash based programming" OR "cash based response*" OR "financial assistance" OR "financial benefit*" OR "financial measure*" OR "financial allowance*" OR "financial payment*" OR "monetary benefit*" OR "monetary transfer*" OR "monetary allowance*" OR "monetary payment*" OR "money allowance*" OR "money transfer*" OR "money voucher*" OR "multi purpose cash" OR "payment measure*" OR "pay for performance" OR "financial incentive*" OR "economic incentive*" OR "monetary incentive*" OR "monetary reimbursement*" OR "reimbursement Incentive*" OR "voucher program*" OR "voucher incentive*")) |
| --- | --- |
| #2 | MAINSUBJECT.EXACT("Overweight") OR MAINSUBJECT.EXACT("Obesity") OR MAINSUBJECT.EXACT("Body mass index") OR MESH.EXACT.EXPLODE("Overweight:E.01.370.600.115.100.160.120.699") OR MESH.EXACT.EXPLODE("Obesity:C.23.888.144.699.500") OR MESH.EXACT.EXPLODE("Obesity:G.07.100.100.160.120.699.500") OR MESH.EXACT.EXPLODE("Obesity:E.01.370.600.115.100.160.120.699.500") OR MESH.EXACT.EXPLODE("Obesity:C.18.654.726.500") OR MESH.EXACT.EXPLODE("Waist Circumference:G.07.100.100.160.560") OR MESH.EXACT.EXPLODE("Weight Gain:C.23.888.144.243.926") OR MESH.EXACT.EXPLODE("Body Mass Index:E.01.370.600.115.100.125") OR MESH.EXACT.EXPLODE("Waist Circumference:E.01.370.600.115.100.160.560") OR MESH.EXACT.EXPLODE("Adiposity:E.01.370.600.115.100.062.500") OR MESH.EXACT.EXPLODE("Adiposity:G.03.180.134.500") OR MESH.EXACT.EXPLODE("Waist Circumference:E.05.041.124.160.875") OR MESH.EXACT.EXPLODE("Body Weight:C.23.888.144") OR MESH.EXACT.EXPLODE("Weight Gain:G.07.345.249.314.120.200.926") OR MESH.EXACT.EXPLODE("Body Mass Index:N.06.850.505.200.100.175") OR MESH.EXACT.EXPLODE("Body Weight:E.05.041.124.160.750") OR MESH.EXACT.EXPLODE("Adiposity:G.07.100.049.134.500") OR MESH.EXACT.EXPLODE("Body Mass Index:G.07.100.100.125") OR MESH.EXACT.EXPLODE("Body Mass Index:E.05.041.124.125") OR MESH.EXACT.EXPLODE("Body Weight:G.07.100.100.160.120") OR MESH.EXACT.EXPLODE("Overweight:C.23.888.144.699") OR MESH.EXACT.EXPLODE("Waist-Hip Ratio:G.07.100.100.960") OR MESH.EXACT.EXPLODE("Adiposity:G.02.111.130.134.500") OR MESH.EXACT.EXPLODE("Skinfold Thickness:E.05.041.124.803") OR MESH.EXACT.EXPLODE("Skinfold Thickness:G.07.100.100.803") OR MESH.EXACT.EXPLODE("Body Weight:E.01.370.600.115.100.160.120") OR MESH.EXACT.EXPLODE("Overweight:G.07.100.100.160.120.699") OR MESH.EXACT.EXPLODE("Body Weight:G.07.345.249.314.120") OR MESH.EXACT.EXPLODE("Skinfold Thickness:E.01.370.600.115.100.803") OR noft(("overweight" OR "overnutrition" OR "over nutrition" OR "obesity" OR "obese" OR "body mass index" OR "BMI" OR "body weight*" OR "waist circumference*" OR "waist-to-hip ratio*" OR "waist-to-height ratio*" OR "skinfold thickness" OR "skin fold thickness" OR "skin fold measurement*" OR "skinfold measurement*" OR "weight gain*" OR "adiposity")) |
| #3 | #1 AND #2 |

**Scopus**

**Date run: 8Aug2021: 564 results**

| #1 | TITLE-ABS-KEY ("cash allowance*" OR "cash assistance" OR "cash benefit*" OR "cash measure*" OR "cash payment*" OR "cash transfer*" OR "cash based approach*" OR "cash based assistance" OR "cash based programming" OR "cash based response*" OR "financial assistance" OR "financial benefit*" OR "financial measure*" OR "financial allowance*" OR "financial payment*" OR "monetary benefit*" OR "monetary transfer*" OR "monetary allowance*" OR "monetary payment*" OR "money allowance*" OR "money transfer*" OR "money voucher*" OR "multi purpose cash" OR "payment measure*" OR "pay for performance" OR "financial incentive*" OR "economic incentive*" OR "monetary incentive*" OR "monetary reimbursement*" OR "reimbursement Incentive*" OR "voucher program*" OR "voucher incentive*") |
| --- | --- |
| #2 | TITLE-ABS-KEY ("overweight" OR "overnutrition" OR "over nutrition" OR "obesity" OR "obese" OR "body mass index" OR "BMI" OR "body weight*" OR "waist circumference*" OR "waist-to-hip ratio*" OR "waist-to-height ratio*" OR "skinfold thickness" OR "skin fold thickness" OR "skin fold measurement*" OR "skinfold measurement*" OR "weight gain*" OR "adiposity") |
| #3 | #1 AND #2 |

**Web of Science**

**Date run: 8Aug2021: 590 results**

| #1 | TS=("cash allowance*" OR "cash assistance" OR "cash benefit*" OR "cash measure*" OR "cash payment*" OR "cash transfer*" OR "cash based approach*" OR "cash based assistance" OR "cash based programming" OR "cash based response*" OR "financial assistance" OR "financial benefit*" OR "financial measure*" OR "financial allowance*" OR "financial payment*" OR "monetary benefit*" OR "monetary transfer*" OR "monetary allowance*" OR "monetary payment*" OR "money allowance*" OR "money transfer*" OR "money voucher*" OR "multi purpose cash" OR "payment measure*" OR "pay for performance" OR "financial incentive*" OR "economic incentive*" OR "monetary incentive*" OR "monetary reimbursement*" OR "reimbursement Incentive*" OR "voucher program*" OR "voucher incentive*") |
| --- | --- |
| #2 | TS= ("overweight" OR "overnutrition" OR "over nutrition" OR "obesity" OR "obese" OR "body mass index" OR "BMI" OR "body weight*" OR "waist circumference*" OR "waist-to-hip ratio*" OR "waist-to-height ratio*" OR "skinfold thickness" OR "skin fold thickness" OR "skin fold measurement*" OR "skinfold measurement*" OR "weight gain*" OR "adiposity") |
| #3 | #1 AND #2 |

**WHO Global Index Medicus**

**Date run: 8Aug2021: 31 results**

<https://www.globalindexmedicus.net/>

(MH:N03.219.521.710.305.380$ OR "Reimbursement Incentive" OR "Reimbursement Incentives" OR "Reembolso de Incentivo" OR "Reembolso de Incentivo" OR MH:N03.219.483$ OR MH:F02.463.425.770.836.500$ OR "cash allowance" OR "cash allowances" OR "cash assistance" OR "cash benefit" OR "cash benefits" OR "cash measure*" OR "cash measures" OR "cash payment*" OR "cash payments" OR "cash transfer*" OR "cash transfers" OR "cash-based approach*" OR "cash-based approaches" OR "cash-based assistance" OR "cash-based programming" OR "cash-based response*" OR "cash-based responses" OR "financial assistance" OR "financial benefit*" OR "financial benefits" OR "financial measure*" OR "financial measures" OR "financial allowance" OR "financial allowances" OR "financial payment" OR "financial payments" OR "monetary benefit" OR "monetary benefits" OR "monetary transfer" OR "monetary transfers" OR "monetary allowance" OR "monetary allowances" OR "monetary payment" OR "monetary payments" OR "money allowance" OR "money allowances" OR "money transfer" OR "money transfers" OR "money voucher" OR "money vouchers" OR "multi-purpose cash" OR "payment measure" OR "payment measures" OR "pay for performance" OR "financial incentive" OR "financial incentives" OR "economic incentive" OR "economic incentives" OR "monetary incentive" OR "monetary incentives" OR "monetary reimbursement" OR "monetary reimbursements" OR "voucher program" OR "voucher programs" OR "voucher incentive" OR "voucher incentives") AND (MH:C18.654.726.500$ OR MH:C23.888.144$ OR MH:E01.370.600.115.100.062.500$ OR MH:E01.370.600.115.100.160.120$ OR MH:E01.370.600.115.100.160.560$ OR MH:E01.370.600.115.100.803$ OR MH:E01.370.600.115.100.960$ OR MH:E05.041.124.125$ OR MH:E05.041.124.160.750$ OR MH:E05.041.124.160.875$ OR MH:E05.041.124.803$ OR MH:E05.041.124.946$ OR MH:G02.111.130.134.500$ OR MH:G03.180.134.500$ OR MH:G07.100.049.134.500$ OR MH:G07.100.100.160.120$ OR MH:G07.100.100.160.560$ OR MH:G07.100.100.803$ OR MH:G07.100.100.960$ OR MH:G07.345.249.314.120$ OR MH:N06.850.505.200.100.175$ OR MH:SP6.011.042.048.024$ OR MH:SP6.011.042.048.029$ OR MH:SP6.011.042.048.054$ OR MH:SP6.011.042.048.069$ OR MH:SP6.011.042.048.079$ OR MH:SP6.016.047$ OR "overweight" OR "overnutrition" OR "over nutrition" OR "obesity" OR "obese" OR "body mass index" OR "BMI" OR "body weight" OR "body weights" OR "waist circumference" OR "waist circumferences" OR "waist-to-hip ratio" OR "waist-to-hip ratios" OR "waist-to-height ratio" OR "waist-to-height ratios" OR "skinfold thickness" OR "skin fold thickness" OR "skin fold measurement" OR "skin fold measurements" OR "skinfold measurement" OR "skinfold measurements" OR "weight gain" OR "weight gains" OR "adiposity")
