# Supplementary material for: Relationship of cash transfers with risk of overweight and obesity in children and adults: a systematic review
Source: BMC Public Health. 2022 Jun 15;22:1190. doi: 10.1186/s12889-022-13533-x (PMC9198205; doi:10.1186/s12889-022-13533-x)
Supplement: Supplementary file 2 — Additional file 2: Supporting Table 2. Characteristics of specific cash transfer programs. [file 12889_2022_13533_MOESM2_ESM.docx]

**Supporting Table 2. Characteristics of specific cash transfer programs**

| **Country** | **Program** | **Type** | **Beneficiaries** | **Conditions** | **Structure** | **Reference** |
| --- | --- | --- | --- | --- | --- | --- |
| Mexico | Originally *Progresa* (1997-2002)*, Oportunidades* (2002-2014), then *Próspera*; program abolished in 2019 | CCT | households with estimated per capita income lower than minimum to acquire basic food basket | household members attending health services, educational communication activities, school attendance for children | CT every 2 months; amount varies by household composition | 33 |
| Colombia | *Más Familias en Acción* | CCT | poor and vulnerable families that have members <18 y; eligibility determined by score in social program system | monitoring growth and development for children <7 y; school attendance children 6-18 y | CT every 2 months; amount varies by location, number and age of children | 34 |
| Peru | *Juntos* | CCT | poor families with children <14 y or a pregnant woman; | regular health care visits for children <5 y or pregnant/lactating women; school attendance for children 6-14 y | CT every month of 100 soles (~US $30) regardless of household composition | 18 |
| Brazil | *Bolsa família* | CCT | families living in poverty (per capita incomes <R$154 in 2015); standards established by national secretariat; eligibility determined by municipality | vaccinations and monitoring of growth and development for children <7 y; prenatal care for pregnant women and lacting mothers; school attendance for children 6-17 y | CT every month; amount varies by household composition and income | 35 |
| Dominican Republic | *Solidaridad* | CCT | families living in poverty with children, pregnant women, or older adults | health controls for children <5 y; ID documents for all family members; parents attend health talks and workshops | CT of RD$ 700 per month (~US $18.40 per month) per family [in 2011] | 36 |
| Mexico | *Reconocer* | UCT | adults ≥70 y in rural areas of Yucatan state, Meico | --- | CT of MXN $550/month (~US$87/month at 2014 purchasing power parity), equivalent to 44% increase in average household income | 26, 37 |
| South Africa | Child Support Grant | UCT | families with children <18 y with annual income below threshold; applicant and spouse must meet requirements of means testing | --- | CT every month | 21 |
| USA | Alaska Permanent Fund Dividend | UCT | Alaska residents | --- | annual universal payment from Permanent Fund Dividend, has averaged $1600/person over last two decades | 23 |
| Canada | Universal Child Care Benefit | UCT | low-income families with children <6 y | --- | CT $CA 100 every month | 31 |
| Japan | *Jido teate* | UCT | households with children from birth-15 y with household income below threshold | --- | CT every month; amount varies by child age and household income | 20 |

Abbreviations: CCT, conditional cash transfer; UCT, unconditional cash transfer
